# Efficacy of Dual Hormonal Therapy with Fulvestrant and Aromatase Inhibitors as Neoadjuvant Endocrine Treatment for Locally Advanced Breast Cancer

**DOI:** 10.3390/cancers17132083

**Published:** 2025-06-21

**Authors:** Ana Majić, Žarko Bajić, Marija Ban, Ivana Tica Sedlar, Dora Čerina Pavlinović, Branka Petrić Miše, Ante Strikić, Snježana Tomić, Eduard Vrdoljak

**Affiliations:** 1 Department of Oncology, University Hospital Center Split, School of Medicine, University of Split, 21000 Split, Croatia; majicana88@gmail.com (A.M.); marija.ban1979@gmail.com (M.B.); tica1978@gmail.com (I.T.S.); dora09cerina@gmail.com (D.Č.P.); brapemi@gmail.com (B.P.M.); astrikic@gmail.com (A.S.); 2 Research Unit “Dr. Mirko Grmek”, Psychiatric Clinic Sveti Ivan, 10090 Zagreb, Croatia; zarko@biometrika.hr; 3 Department of Pathology, Forensic Medicine and Cytology, Clinical Hospital Center Split, School of Medicine, University of Split, 21000 Split, Croatia; st@mefst.hr

**Keywords:** breast neoplasms, neoadjuvant therapy, endocrine therapy, fulvestrant, aromatase inhibitors, Ki-67 antigen, real-world evidence

## Abstract

**Background**: The role of neoadjuvant endocrine therapy (NET) in patients with luminal tumors is still not well defined in everyday clinical practice. To assess the efficacy of combination NET, we analyzed the outcomes of fulvestrant and aromatase inhibitors (AI) in combination in a real-world population. **Methods**: This was a single-arm, retrospective longitudinal study of the total population of patients diagnosed with locoregionally advanced, clinical stage II-III, HR+ HER2-, luminal-type eBC, who were treated with the neoadjuvant combination of fulvestrant and AI between 2019 and 2024 at the Clinical University Hospital of Split, Croatia. **Results**: We enrolled 44 patients in the intention-to-treat (ITT) population, while 34 completed NET and surgery (per-protocol population; PPP). The median duration of NET was 11 months (interquartile range [IQR] of 9–16 months). The best radiological objective response rate (partial or complete response) was achieved by 30 (68.2%) in ITT, and 26 (76.5%) in PPP, defined by radiological examination, breast ultrasound, or MR. In the PPP, the minimal or moderate pathological response according to residual cancer burden (I or II) was observed in 29 (85.3%) patients. The median of absolute changes in Ki-67 was −5 (95% CI: −9 to 0), and the median of relative Ki67 changes was −40% (95% CI: −72% to 0%). Post-surgical Ki-67 was significantly predicted by initial Ki-67, positive lymph nodes, and time from diagnosis to the initiation of NET. Treatment was well tolerated, with no therapy discontinuation or dose reductions needed due to toxicity. The most commonly reported side effects included musculoskeletal pain (45.5%), asthenia (34.1%), and hot flashes (29.5%). **Conclusions**: Dual hormonal therapy with fulvestrant and AI is an active, easily given, non-toxic, promising neoadjuvant treatment in real-world patients with locally advanced luminal-type eBC who are not candidates for chemotherapy.

## Simple Summary

Some breast cancers grow in response to hormones such as estrogen. In certain patients, especially older women or those with additional health issues, traditional chemotherapy may not be the best option for neoadjuvant therapy. Instead, hormone-blocking treatments can be given before surgery to shrink the tumor and make the operation easier. This study looked at the use of two hormone therapies—fulvestrant and an aromatase inhibitor—used together before surgery in women with advanced breast cancer. Most patients responded well, with tumor shrinkage and fewer cancer cells visible after treatment. The therapy was also well tolerated, with few side effects. These findings suggest that this dual hormone treatment may be a promising option for patients who are not good candidates for chemotherapy and could help improve outcomes while reducing treatment-related risks.

## 1. Introduction

Breast cancer is a leading health concern due to its high mortality and morbidity rates s. In 2022, 2.3 million new breast cancer cases and 670,000 deaths occurred globally. Breast cancer is the most common cancer among Croatian women, accounting for 26% of female cancer cases, with about 3100 cases in 2022 [1,2].

Unfortunately, there is still a large number of patients who initially present with locally advanced or metastatic disease despite the implementation of early detection programs. Neoadjuvant therapy (NAT) was initially introduced to treat locally advanced, borderline operable, or inoperable diseases to make it more accessible for surgery and to allow for breast-conserving surgery (BCS). Nowadays, after the results of clinical trials have shown that NAT is equally as effective as adjuvant therapy in terms of overall survival and disease-free survival, the usage of NAT has widened to earlier stages of disease, especially in the case of triple-negative and HER-2 positive cancers [3]. Pathological complete response (pCR) rates of about 65% are expected when triple-negative (TNBC) and HER2-positive (HER2+) tumors are treated with neoadjuvant therapy [4,5]. The outcomes of neoadjuvant chemotherapy (NACT) in luminal A or B tumors are much less pronounced than with HER-2+ + or TNBC, with pCR rates of less than 10% [6]. Consequently, careful patient selection for NACT is crucial for successful outcomes in luminal tumors. Moreover, many patients who need NACT are not ideal candidates for it due to their general status, age, or comorbidities. Neoadjuvant endocrine therapy (NET) is a valid treatment option for some luminal breast cancers, especially in postmenopausal women, as an alternative to chemotherapy. It offers similar rates of breast-conserving surgery with fewer side effects [7]. Consequently, NET is considered the treatment of choice for patients in need of tumor downstaging and luminal A or low-risk luminal B tumors, especially for elderly and patients with significant comorbidities. Results from clinical trials and meta-analysis have confirmed the position of aromatase inhibitors (AI) as a gold standard for NET in postmenopausal women [8,9,10,11]. Fulvestrant, a selective ER degrader (SERD), statistically significantly and clinically meaningfully improved outcomes in terms of progression-free survival (PFS) and overall survival (OS) in the metastatic setting in combination with AI, based on the results of SWOG S0226, and this questioned the role of such a combination in the early, neoadjuvant setting [12]. The selection of fulvestrant and aromatase inhibitors (AIs) in our study was driven by their complementary mechanisms of action and prior evidence suggesting synergistic efficacy in hormone receptor-positive (HR+) metastatic breast cancer. While an AI reduces systemic estrogen levels by inhibiting aromatase, the enzyme that converts androgens into estrogens, fulvestrant (SERD) binds to and degrades estrogen receptors (ERs), blocking estrogen signaling and reducing ER-driven tumor growth and potentially prevents ER reactivation that can occur with AI monotherapy. This dual blockade targets both estrogen production (via AI) and ER signaling (via fulvestrant), addressing two pathways critical for HR+ tumor survival. Consequently, in ER+ breast cancer models, fulvestrant combined with AIs achieved greater ER suppression than either agent alone, reducing ER levels by 39–41% vs. 13% with anastrozole alone [13,14]. Nevertheless, the recently published trials failed to confirm the superiority of the fulvestrant AI combination to AI alone in neoadjuvant settings [15,16,17]. Our study aims to explore whether fulvestrant combined with AI has the potential to improve the outcomes of patients with locally advanced early breast cancer in a real-world setting and the effect of the duration of the therapy on outcomes.

## 2. Materials and Methods

### 2.1. Study Design

We conducted a single-arm, retrospective longitudinal study on the total population of patients with locoregionally advanced luminal-type breast cancer diagnosed during 2019–2024 and treated with NET at the Clinical University Hospital of Split, Croatia. The study was approved by the institutional ethics committee [18]. Informed consent was obtained from all participants prior to data retrieval. Before analysis, all records were anonymized. The study adhered to the ethical principles outlined in the Declaration of Helsinki (1975, revised 2013) [18]. As this was a retrospective study, the protocol was not registered in advance, and no central review of imaging or pathology data was performed. Clinical information was extracted from institutional electronic health records by the first author using a predefined and uniform data collection procedure.

### 2.2. Participants

The study population consisted of women diagnosed with locally advanced luminal breast cancer who received NET consisting of fulvestrant and AI in the period from May 2019 until January 2024, taken as the time we started using such a combination in everyday clinical practice. Eligible patients were female, diagnosed with locally advanced ER+, HER2 breast cancer, confirmed by core needle biopsy. The pretreatment evaluation included breast ultrasound and mammography. Tumor response was evaluated clinically and radiologically throughout the treatment period. At the end of the planned treatment, the patients had surgery if they were eligible, and the pathological results of posttreatment samples were analyzed to define a pathological response. All patients were considered poor candidates for neoadjuvant chemotherapy based on advanced age, comorbidities, poor performance status, tumor characteristics (e.g., low proliferative index), or personal preference. None received adjuvant chemotherapy after surgery. For the patients who were not candidates for surgery or declined surgery, clinical response to therapy was evaluated at the planned data cut-off. The sample was not selected but encompassed all patients who received neoadjuvant therapy with fulvestrant and an aromatase inhibitor during the study period. As the aim was to include the entire eligible population, a pre-study power analysis was not conducted.

### 2.3. Endpoints

The primary endpoint was the efficacy of dual hormonal combination therapy with fulvestrant and AI, measured by the pathological response. The secondary efficacy endpoints were changes in Ki-67 values, as well as clinical and radiological responses. Secondary safety outcomes included the frequency of adverse events related to treatment, classified as hematological or non-hematological, across all severity levels, as well as those classified as grade 3 or 4. We also assessed the proportion of patients who experienced dose reductions, temporary interruptions, or permanent discontinuation of therapy due to toxicity. Adverse event grading followed the Common Terminology Criteria for Adverse Events, version 5.0.

### 2.4. Treatment

The patients received fulvestrant 500 mg IM on days 1, 15, and 29 and once monthly thereafter and an aromatase inhibitor (anastrozole 1 mg daily or letrozole 2.5 mg daily), based on the choice of the treating physician, daily throughout the treatment period. The therapy was administered until the optimal response was determined by the ordering oncologist before planned surgery (minimum duration of 6 months), the disease progressed, or unacceptable toxicity developed.

### 2.5. Statistical Analysis

This study involved a retrospective observational cohort without randomization or a predefined control group. We pragmatically defined the intention-to-treat (ITT) population as all patients who initiated neoadjuvant endocrine therapy (NET), regardless of treatment completion. The per-protocol (PP) population included only those who completed both NET and subsequent surgery. Although the terms ITT and PP are standard in randomized trials, their use here is intended to clearly distinguish between the total treated population and those evaluable for pathological response.

The primary analysis was conducted in the intention-to-treat (ITT) population, which included all patients enrolled in the study, regardless of whether they completed NET and underwent surgery or not. The post-treatment Ki-67 values were missing for ten patients in the ITT population due to the absence of surgical specimens. The missing Ki-67 values for these ten patients were imputed in the ITT population using the initial values measured before the start of NET.

The time-to-event outcomes (e.g., time to best radiological response) are summarized descriptively using medians and interquartile ranges (IQRs). No formal survival analysis was undertaken due to the limited follow-up period and event frequency.

To evaluate predictors of post-surgical Ki-67, the proliferation index was expressed as a percentage. Given that this outcome is continuous, bounded (0–100%), and right-skewed, we applied beta regression with the complementary log-log link function to appropriately model the rates and proportions, excluding the boundary values. This approach was more suitable than linear regression, which assumes unbounded and normally distributed residuals. We reported the average marginal effects (AMEs) for all predictors to provide interpretable effect sizes, expressed as percentage point changes in the predicted post-treatment Ki-67 value for one-unit increases in continuous variables or relative to the reference category for categorical ones.

We used Huber–White robust standard errors to account for potential heteroscedasticity and ensure valid inference under mild model misspecification. The model included baseline Ki-67, age at diagnosis, histological subtype, immunophenotype (luminal A vs. luminal B), clinical T stage, lymph node status, histological grade, estrogen receptor (ER) and progesterone receptor (PR) expression (as continuous variables), time from diagnosis to NET initiation, the specific aromatase inhibitor used (letrozole vs. anastrozole), and whether surgery was performed. The covariates included in the multivariable model were selected a priori based on clinical relevance and evidence from previously published studies. We avoided dichotomizing continuous covariates in multivariable models to retain statistical power and reduce residual confounding due to categorization. Transformations of predictors were not applied, and no interaction terms or hierarchical models were included, given the limited sample size and to avoid overfitting. To assess the predictors of binary outcomes, such as objective radiological response and residual cancer burden class, we used logistic regression models, adjusting for relevant covariates. Due to the small number of events, wide confidence intervals were expected, and the results should be interpreted cautiously. Two-tailed statistical significance was set at *p* < 0.05, and all confidence intervals (CI) were calculated at a 95% level. To reduce the likelihood of false-positive findings due to multiple hypothesis testing in the multivariable models, we applied Benjamini–Hochberg correction with the false discovery rate (FDR) set at 5%. This approach provides a balance between Type I error control and statistical power, which is especially suitable for exploratory multivariable analyses. All statistical analyses were conducted using Stata version 19.0 (Revision 21 May 2025; StataCorp LLC, College Station, TX, USA).

## 3. Results

### 3.1. Patients, Tumor, and Treatment Description

From May 2019 to January 2024, 44 patients with clinical stage II-III HR+ HER2− breast cancer were enrolled in data analysis, of whom 34 (77.3%) had surgery by the data cut-off date and were eligible for pathological response assessment (Table 1). The median age was 74 (IQR 64–79) years, while 39 (88.6%) of patients were postmenopausal. LHRH agonists were used in all five premenopausal women. Node-positive disease was detected in 17 (38.6%) patients. Of the 44 patients who initiated NET (ITT population), 34 completed both NET and surgery (PPP). Among the ten patients who did not proceed to surgery, four experienced disease progression or died during NET, one declined surgery, and five were still undergoing treatment at the time of data cut-off. None of the patients had a baseline Ki-67 index below 2.7%. No patient received postoperative chemotherapy.

The median time from diagnosis to the initiation of NET was 34 (IQR 22–50) days, and its median duration was 11 (IQR 9–16) months, over a median of 12 (IQR 12–17) treatment cycles (Table 2). At the time of analysis, 6/44 (13.6%) patients were still in active NET.

### 3.2. Efficacy and Safety Outcomes

The best radiological response was achieved after a median of 4.7 (IQR 3.4–8.2) months from the initiation of NET. One patient experienced disease progression, and 30 (68.2%) had ORR in the ITT population (Table 3). Four (9.1%) patients in the ITT population had a complete radiological response. Only four patients had breast-conserving surgery, and 30/34 (88.2%) had mastectomies. A pathological assessment of postoperative specimens was performed for all 34 patients, and 29/34 (85.3%) had a residual cancer burden index (RCB) score of I (minimal) or II (moderate). Hematological toxicities were not observed. Non-hematologic adverse events were grade 1–2 and included musculoskeletal pain, asthenia, hot flashes, and injection site reactions. No dose reduction or discontinuations were required. Four deaths occurred during the follow-up period; none were attributed to treatment-related toxicity.

### 3.3. Predictors of Treatment Outcomes

Baseline Ki-67 levels significantly predicted post-surgical Ki-67 values, with an average marginal effect (AME of 0.55 (95% CI 0.27 to 0.84, *p* < 0.001, FDR < 5%) in the fully adjusted multivariable model (Table 4). For every one percentage point increase in the baseline Ki-67, the post-surgical Ki-67 levels increased by 0.55%. The patients with positive lymph nodes had post-surgical Ki-67 levels that were, on average, 4.87% higher (95% CI 0.06 to 9.68, *p* = 0.047, FDR < 5%) compared to those without nodal involvement. Furthermore, each additional month between diagnosis and the initiation of NET was associated with a 1.56% increase in post-surgical Ki-67 levels (95% CI: 0.05 to 3.07, *p* = 0.042, FDR < 5%). Age at diagnosis, histological subtype, immunophenotype (luminal A vs. luminal B), clinical T stage, histological grade, and hormone receptor status (ER and PR) did not independently predict the post-surgical Ki-67 levels in the fully adjusted model. These results were mostly confirmed in the per-protocol population (Table 5). Only tumor grade was found to be statistically significantly associated with ORR, as assessed by multivariate binary logistic regression, but the findings are unreliable (odds ratio of 25.02; 95% CI 2.36; 265.48; *p* = 0.008, FDR < 5%).

## 4. Discussion

The primary goals of NAT in breast cancer are increasing rates of BCS; evaluating tumor response to systemic therapy in vivo, which can provide prognostic information and guide subsequent treatment decisions; improving long-term outcomes, particularly in aggressive subtypes like triple-negative and HER2-positive breast cancers; and providing an opportunity for translational research to identify biomarkers of response and resistance [19,20]. The initial aim of implementing the neoadjuvant approach in treating breast cancer was to increase the rate of breast-conserving surgery (BCS) in patients who initially were not candidates for it. According to available clinical data, BCS after NAT is a feasible and safe option for patients with initial cT3-4. The results reveal that choosing BCS after NAT did not compromise the long-term oncological outcomes compared to mastectomy. Still, the decision between BCS and mastectomy remains multifactorial, including the surgeon’s assessment and the patient’s personal preferences. The proportion of patients in our study who underwent BCS was small, mainly due to the personal choice of patients to have mastectomy instead of BCS. The reason for this might have been the patient’s age or comorbidities, as well as the potential of avoiding radiotherapy in adjuvant treatment [21]. Large clinical data is now available for NAT in HER2+ or TNBC, with clearly defined guidelines available [22,23]. Consequently, neoadjuvant chemoimmunotherapy can be considered a standard in these subtypes of tumors larger than 2 cm in size or with positive axillary lymph nodes [24]. Despite the advantages of endocrine therapy with AI in the neoadjuvant setting, as shown in clinical trials, due to undefined prognostic markers of response, the unclear optimal duration of therapy, and a vague definition of the population of patients to be treated, there is still limited use of NET by physicians in real-world practice. Factors like tumor grade, Ki67 levels, and progesterone receptor status can help to select patients who are more likely to benefit from NACT than NET [7]. The rationale for our study that fulvestrant may enhance the efficacy of AI in a neoadjuvant setting is based on the results of clinical trials for advanced disease settings, which suggested that the addition of fulvestrant to anastrozole was associated with increased long-term survival as compared with anastrozole alone. The FALCON trial, which investigated whether the fulvestrant (500 mg intramuscular injection; on days 0, 14, and 28, and then every 28 days thereafter) could improve progression-free survival compared with anastrozole (1 mg orally daily) in postmenopausal patients who had not received previous endocrine therapy showed that progression-free survival was significantly longer in the fulvestrant group than in the anastrozole group (hazard ratio [HR] 0·797, 95% CI 0.637–0.999, *p* = 0.0486 [25]). The results of this combination therapy used in the first-line metastatic setting showed a risk reduction of death of 18% when compared to anastrazole alone. The median overall survival was 42.0 months in the anastrozole-alone group and 49.8 months in the combination-therapy group, based on 261 and 247 deaths, respectively. The survival difference between the two treatment arms was significant (hazard ratio, 0.82; 95% CI, 0.69 to 0.98; *p* = 0.03 by the log-rank test). In comparison to the mentioned results from the metastatic setting, fulvestrant and anastrozole were investigated in the neoadjuvant setting in the CARMINA02 and HORGEN trials without significant differences in terms of BCS, pathological response, and survival rates. Pooled analysis of these trials showed that relapse-free survival (RFS) and overall survival (OS) at 5 years were 83.7% (95% CI: 77.9; 88) and 92.7% (95% CI: 88.2; 95.6), respectively, with no difference between the treatment arms. [15,16]. The results from the ALTERNATE clinical trial, unfortunately, also failed to confirm the superiority of the fulvestrant combination regimen [17]. In our study, we aimed to investigate the potential of this dual therapy in the real-world setting. Our primary endpoint, pathological response based on pathological results, showed that 85.3% of patients had a residual cancer burden index (RCB) score of I (minimal) or II (moderate). These results are consistent with the results from clinical trials [26]. Unlike in other breast cancer subtypes, the correlation between survival outcome and pCR is weaker in luminal types of breast cancer [27].

There is an unmet need for the development of new biomarkers that could be used for the determination of outcomes with NET, especially in comparison with NACT. Nowadays, the preoperative endocrine prognostic index (PEPI) score and Ki67 measurements before or after surgery are widely used [28]. Tumors that show substantial down-staging after NET and present with low Ki67 levels and a low PEPI score have an excellent long-term prognosis [11]. As it is known that fulvestrant can reduce ER levels, in our analysis, we used the mPEPI score used in the previously mentioned ALTERNATE trial, which excludes the ER component (Allred score). The mPEPI score remains prognostic despite the exclusion of ER, as it includes other relevant prognostic factors, like tumor size, node involvement, and Ki67 value. The final score was determined by summing the risk points derived from the tumor characteristics of the surgical specimen after neoadjuvant treatment. An mPEPI of pT1/2, pN0, Ki67 ≤ 2.7% was clinically equivalent to a PEPI score zero, and those tumors showed a very low probability of recurrence [29,30].

The combination of fulvestrant and AI inhibited tumor proliferation, as measured by the decrease in the Ki67 level. Clinical response rates, evaluated radiologically, were high across our patients, with 68.2% achieving ORR in the ITT population. The optimal duration of NET is still undefined, and this raises the question of whether the short duration of NET may be the reason behind the lower response rates observed compared with those when chemotherapy is used [31]. Clinical trials evaluating the efficacy of NACT showed pCR rates from 10 to 20%, depending mostly on tumor subtype, and therapy is often given for a total of 3 to 6 months, depending on the drugs used [32,33]. In comparison to chemotherapy, NET has a more gradual effect on the tumor, and a longer treatment period is usually required to reach the maximum clinical response. One of the major concerns of extending NET until maximal response is the risk of disease progression, so the careful monitoring of disease during treatment is crucial [34]. In our study, the duration of therapy was not fixed in advance but was left to the discretion of the treating physician, and the results show that a longer duration of therapy resulted in better clinical outcomes, with 56.8% of patients experiencing an improved radiological response. While our data suggests an association between longer NET duration and improved responses, causality cannot be established due to the non-randomized, observational design. In contrast to the ALTERNATE trial, in which radiological response rates were not analyzed since pretreatment and posttreatment imaging were completed in less than 60% of patients, all of our patients had radiological evaluation before, during, and after the planned treatment. Our secondary endpoint analysis of radiological or clinical response showed high radiological response rates, with a rate of complete response of 9.1%. The results of the P024 trial showed an ORR of 52% with letrozole alone. The IMPACT trial, comparing anastrozole, tamoxifen, and their combination, had clinical response rates of around 35% and ultrasound response rates of around 25%, without significant differences between the hormonal agents used. Our analysis showed that adding fulvestrant to AI resulted in an ORR of 68.2% in an intention-to-treat population. In the era of new drugs, such as CDK4/6 inhibitors, with proven efficacy in metastatic settings in combination with fulvestrant or aromatase inhibitors, their role in neoadjuvant settings has been widely investigated, and early clinical data on the neoadjuvant treatment of hormone receptor-positive/HER2-negative breast cancer is promising [35,36,37,38]. When considering the high drug costs and potential for adverse effects, there is a question of the cost effectiveness of using them in neoadjuvant treatment, especially in low-income countries.

Treatment was generally well tolerated with no discontinuations or dose reductions, and only mild to moderate adverse events were reported, aligning with safety data from prior fulvestrant trials [39].

### Limitations of the Study

The first limitation of our study is the lack of a control group, which restricts our ability to draw valid and robust conclusions regarding the efficacy of the combination NET compared to other treatment options, including neoadjuvant AI monotherapy. The second key limitation is the small number of patients included in the analysis, attributable to the limited available population during the enrolment period. A consequence of the small sample size is the reduced statistical power and an increased risk of false negative findings in the analysis of predictors of treatment response. Additionally, the small cohort limits the reliability, precision, and external validity (generalizability) of the results, although this does not necessarily invalidate the study’s overall validity.

Another important limitation is the absence of centralized pathology and imaging review, which may introduce inter-observer variability and subjective bias in the assessment of treatment response. Although all evaluations were performed by experienced institutional specialists following standard protocols, the lack of independent verification reduce the methodological rigor and the interpretability of the response outcomes.

Despite these limitations, our study presents a real-world, institutional experience based on all consecutive patients treated with this regimen, The results suggest the potential value of the combination of fulvestrant and AI as a NET strategy in daily clinical practice for patients with advanced luminal-type breast cancer.

## 5. Conclusions

In real-world settings, dual hormonal therapy with fulvestrant and AI is potentially an effective combination in the neoadjuvant treatment of luminal breast cancer. Our findings, derived from an unselected patient population, show promising clinical efficacy and good tolerability of this neoadjuvant therapy protocol in patients with locally or locoregionally advanced luminal breast cancer. Although clinical trial evidence is primarily based on data from postmenopausal patients, our results suggest that this less aggressive treatment may be an option for younger, premenopausal patients as well.

In an era of expensive new drugs being investigated in neoadjuvant settings for luminal tumors, fulvestrant is a cost-effective option that provides high clinical response rates when added to standard aromatase inhibitor therapy, making it a valuable treatment option, especially in healthcare systems with limited resources. The general unavailability of multigene breast cancer prognostic tests in many healthcare systems is an additional limiting factor in selecting patients who may not benefit from neoadjuvant chemotherapy. Consequently, all patients not suitable for NACT due to their tumor or patient characteristics should opt for NAT as an equally effective and less toxic neoadjuvant treatment option for patients in need. Optimal NET should be individualized according to the patients and tumor characteristics; the length of NET, as well as the protocol of therapy should be defined to suit the patient’s needs best. The combination of an AI and fulvestrant is a valid NET option with a good efficacy-to-tolerability ratio. Defining precise follow-up procedures during the treatment and implementing measurements of the Ki67 values after a lead-out time of 1–2 months of NAT would probably result in a better selection of patients and thus to better treatment outcomes. Larger studies and longer follow-ups are needed to report more reliable and reproducible clinical outcomes and to confirm the results of this analysis.

## Figures and Tables

**Table 1:** Patient and tumor characteristics at baseline, before the introduction of neoadjuvant dual hormonal therapy.

	Intention-to-Treat Population (n = 44)	Per-Protocol Population (n = 34)
Age at diagnosis (years), median (IQR)	74 (64–79)	74 (61–77)
Categorized age at diagnosis (years)		
<65	14 (31.8)	13 (38.2)
65–74	16 (36.4)	11 (32.4)
≥75	14 (31.8)	10 (29.4)
Menopausal status		
Premenopause	5 (11.4)	4 (11.8)
Postmenopause	39 (88.6)	30 (88.2)
Histological subtype		
Lobular	15 (34.1)	13 (38.2)
Mucinous	2 (4.6)	1 (2.9)
NOS	27 (61.4)	19 (55.9)
Immunophenotype		
Luminal A	27 (61.4)	22 (64.7)
Luminal B	17 (38.6)	12 (35.3)
Clinical T stage		
T1	1 (2.3)	1 (2.9)
T2	20 (45.5)	19 (55.9)
T3	3 (6.8)	2 (5.9)
T4	20 (45.5)	12 (35.3)
Clinical N stage		
N0	27 (61.4)	24 (70.6)
N1	16 (36.4)	9 (26.5)
N2	1 (2.3)	1 (2.9)
Positive lymph nodes	17 (38.6)	10 (29.4)
Histological grade		
G1	11 (25.0)	8 (23.5)
G2	30 (68.2)	24 (70.6)
G3	3 (6.8)	2 (5.9)
Estrogen receptors (%), median (IQR)	100 (95–100)	100 (94–100)
Progesterone receptors (%), median (IQR)	92 (40–99)	92 (40–99)
Ki-67 index, median (IQR)	13 (9–20)	14 (9–20)
Categorized Ki-67 index		
≤10	16 (36.4)	13 (38.2)
11–20	18 (40.9)	13 (38.2)
21–30	7 (15.9)	6 (17.6)
>30	3 (6.8)	2 (5.9)

Data are presented as numbers (percentages) of patients unless otherwise specified. Abbreviations: IQR, interquartile range; NOS, not otherwise specified.

**Table 2:** Treatment.

	Intention-to-Treat Population (n = 44)	Per-Protocol Population (n = 34)
Time from diagnosis to NET (days), median (IQR)	34 (22–50)	37 (24–50)
Duration of NET (months), median (IQR)	11 (9–16)	10 (9–15)
Number of treatment cycles, median (IQR)	12 (12–17)	12 (11–14)
NET completed	38 (86.4)	32 (100.0)
Specific AI		
Letrozole	32 (76.2)	26 (76.5)
Anastrozole	10 (23.8)	8 (23.5)
Treatment with LHRH	5 (11.4)	4 (11.8)
Surgery	34 (77.3)	34 (100.0)
Time from diagnosis to surgery (months), median (IQR)	12.8 (11.0–16.0)	12.8 (11.0–16.0)
Surgery type *		
Breast-conserving	n.r.	4 (11.8)
Mastectomy	n.r.	30 (88.2)
Adjuvant therapy *		
Endocrine	n.r.	34 (100.0)
Radiotherapy	n.r.	28 (82.4)
Duration of follow-up (months), median (IQR)	24 (16–35)	23 (15–35)

Data are presented as numbers (percentages) of patients unless otherwise specified. Abbreviations: NET, neoadjuvant endocrine treatment; IQR, interquartile range; AI, aromatase inhibitors; LHRH, luteinizing hormone-releasing hormone agonists; n.r., not relevant, and the same as in per-protocol population. * Denominator: Patients who completed NET and underwent surgery (n = 34).

**Table 3:** Treatment outcomes and toxicity.

	Intention-to-Treat Population (n = 44)	Per-Protocol Population (n = 34)
Time from introduction of NET to the best clinical response (months), median (IQR)	3.1 (2.3–8.9)	3.0 (2.3–6.7)
Best clinical response		
CR	0 (0.0)	0 (0.0)
PR	21 (47.7)	13 (38.2)
SD	23 (52.3)	21 (61.8)
PD	0 (0.0)	0 (0.0)
Best clinical objective response	21 (47.7)	13 (38.2)
Time from introduction of NET to the best radiological response (months), median (IQR)	4.7 (3.4–8.2)	5.2 (3.5–8.5)
Best radiological response		
CR	4 (9.1)	4 (11.8)
PR	26 (59.1)	22 (64.7)
SD	13 (29.6)	7 (20.6)
PD	1 (2.3)	1 (2.9)
Best radiological objective response	30 (68.2)	26 (76.5)
Death	4 (9.1)	2 (5.9)
Residual cancer burden index (RCB)	n.a.	
RCB I (minimal)		3 (8.8)
RCB II (moderate)		26 (76.5)
RCB III (extensive)		5 (14.7)
mPEPI score		
0 (very low risk)	n.a.	3 (8.8)
1–3 (intermediate risk)	n.a.	15 (44.1)
≥4 (high risk)	n.a.	16 (47.1)
Ki-67 index changes from baseline, before the introduction of NET, to after the surgery	n.a.	
Median (IQR) of absolute changes		−5 (−9–0)
Median (IQR) of relative changes		−40% (−72–0%)
Patients whose Ki-67 was lowered from >10% to ≤10%	n.a.	12/21 (57.1%)
Patients whose Ki-67 was lowered from >10% to <2.7%	n.a.	3/21 (14.3%)
Ki-67 index < 2.7%	n.a.	7 (20.6%)
Non hematologic toxicity		
Musculoskeletal pain	20 (45.5)	n.a.
Asthenia	15 (34.1)	n.a.
Hot flashes	13 (29.5)	n.a.
Injection site reaction	7 (15.9)	n.a.

Data are presented as numbers (percentages) of patients unless otherwise specified. Abbreviations: NET, neoadjuvant endocrine treatment; IQR, interquartile range; CR, complete response; PR, partial response; SD, stable disease; PD, progressive disease; objective response rate, complete response + partial response; n.a., not available for patients who did not undergo surgery. Relative change in Ki-67 index for each patient was calculated as [(Ki-67 value after the surgery − Ki-67 value at baseline, before the NET)/Ki-67 value at baseline, before the NET] × 100.

**Table 4:** Predictors of Ki-67 results after the surgery adjusted for the baseline Ki-67; in intention-to-treat population (n = 44).

	Adjusted Only for Initial Ki-67			Multivariable, Fully Adjusted		
	AME	(95% CI)	*p*	AME	(95% CI)	*p*
Initial Ki-67 (%)	0.46	(0.19; 0.73)	0.001 *	0.55	(0.27; 0.84)	<0.001 *
Age at diagnosis (years)	0.11	(−0.04; 0.27)	0.159	0.10	(−0.13; 0.33)	0.410
Histological subtype						
Other or NOS	Referent			Referent		
Lobular	−0.38	(−4.22; 3.47)	0.848	0.35	(−3.96; 4.65)	0.875
Immunophenotype						
Luminal A	Referent			Referent		
Luminal B	1.34	(−3.37; 6.04)	0.578	−4.05	(−14.28; 6.19)	0.438
Clinical T stage						
T1 od T2	Referent			Referent		
T3 or T4	−0.26	(−5.67; 5.16)	0.926	−5.05	(−11.50; 1.40)	0.125
Positive lymph nodes	4.60	(−0.03; 9.22)	0.051	4.87	(0.06; 9.68)	0.047 *
Histological grade						
G1	Referent			Referent		
G2 or G3	−3.47	(−8.13; 1.19)	0.144	−0.10	(−6.90; 4.89)	0.739
Estrogen receptors (%)	0.07	(−0.31; 0.44)	0.724	0.11	(−0.46; 0.68)	0.705
Progesterone receptors (%)	−0.02	(−0.07; 0.03)	0.424	−0.04	(−0.14; 0.05)	0.387
Months from diagnosis to introduction of neoadjuvant hormonal therapy	0.84	(−1.08; 2.75)	0.392	1.56	(0.05; 3.07)	0.042 *
Specific AI						
Letrozole	Referent			Referent		
Anastrozole	2.40	(−1.83; 6.63)	0.266	−0.10	(−5.15; 4.95)	0.970
Surgery	−6.84	(−10.47; −3.21)	<0.001 *	−5.96	(−10.91; −1.01)	0.018 *

Abbreviations: AME, average marginal effect, which indicates the change in the expected value of the Ki-67 after the surgery for a one unit increase in each predictor; CI, confidence interval; p, statistical significance of the AME calculated using beta regression with complementary log-log link and robust standard errors estimated using the Huber–White sandwich estimator; AI, aromatase inhibitors. * False discovery rate < 5%.

**Table 5:** Predictors of Ki-67 results after the surgery adjusted for the baseline Ki-67 in the per-protocol population (n = 34).

	Adjusted Only for Initial Ki-67			Multivariable, Fully Adjusted		
	AME	(95% CI)	*p*	AME	(95% CI)	*p*
Initial Ki-67 (%)	0.32	(−0.12; 0.75)	0.151	0.52	(0.13; 0.92)	0.010 *
Age at diagnosis (years)	0.13	(−0.01; 0.28)	0.073	0.15	(−0.12; 0.43)	0.273
Histological subtype						
Other or NOS	Referent			Referent		
Lobular	0.65	(−3.27; 4.56)	0.747	0.92	(−3.35; 5.18)	0.674
Immunophenotype						
Luminal A	Referent			Referent		
Luminal B	1.31	(−2.98; 5.60)	0.548	−5.10	(−15.50; 5.31)	0.337
Clinical T stage						
T1 od T2	Referent			Referent		
T3 or T4	−2.43	(−7.63; 2.78)	0.361	−4.47	(−10.12; 1.18)	0.121
Positive lymph nodes	4.12	(−1.86; 10.09)	0.177	5.88	(1.41; 10.36)	0.010 *
Histological grade						
G1	Referent			Referent		
G2 or G3	−2.89	(−9.62; 3.85)	0.401	−2.61	(−10.16; 4.94)	0.498
Estrogen receptors (%)	0.12	(−0.20; 0.43)	0.462	0.20	(−0.31; 0.72)	0.442
Progesterone receptors (%)	−0.01	(−0.07; 0.05)	0.827	−0.04	(−0.13; 0.05)	0.373
Months from diagnosis to introduction of neoadjuvant hormonal therapy	0.75	(−2.22; 3.71)	0.622	1.35	(−0.52; 3.22)	0.157
Specific AI						
Letrozole	Referent			Referent		
Anastrozole	0.32	(−0.12; 0.75)	0.151	1.49	(−5.47; 8.44)	0.675

Abbreviations: AME, average marginal effect, which indicates the change in the expected value of the Ki-67 after the surgery for a one unit increase in each predictor; CI, confidence interval; p, statistical significance of the AME calculated using beta regression with complementary log-log link and robust standard errors estimated using the Huber-White sandwich estimator; AI, aromatase inhibitors. * False discovery rate < 5%.

## Data Availability

Data and Stata metadata are available from the corresponding author upon the request.

## Abbreviations

The following abbreviations are used in this manuscript:

AI	Aromatase inhibitor
AME	Average marginal effect
BCS	Breast-conserving surgery
CI	Confidence interval
CR	Complete response
eBC	Early breast cancer
ER	Estrogen receptor
FDR	False discovery rate
IQR	Interquartile range
ITT	Intention-to-treat population
NACT	Neoadjuvant chemotherapy
NAT	Neoadjuvant therapy
NET	Neoadjuvant endocrine therapy
NOS	Not otherwise specified
pCR	Pathological complete response
PR	Progesterone receptor
PR	Partial response
RCB	Residual cancer burden index
SD	Stable disease
TNBC	Triple-negative breast cancer
